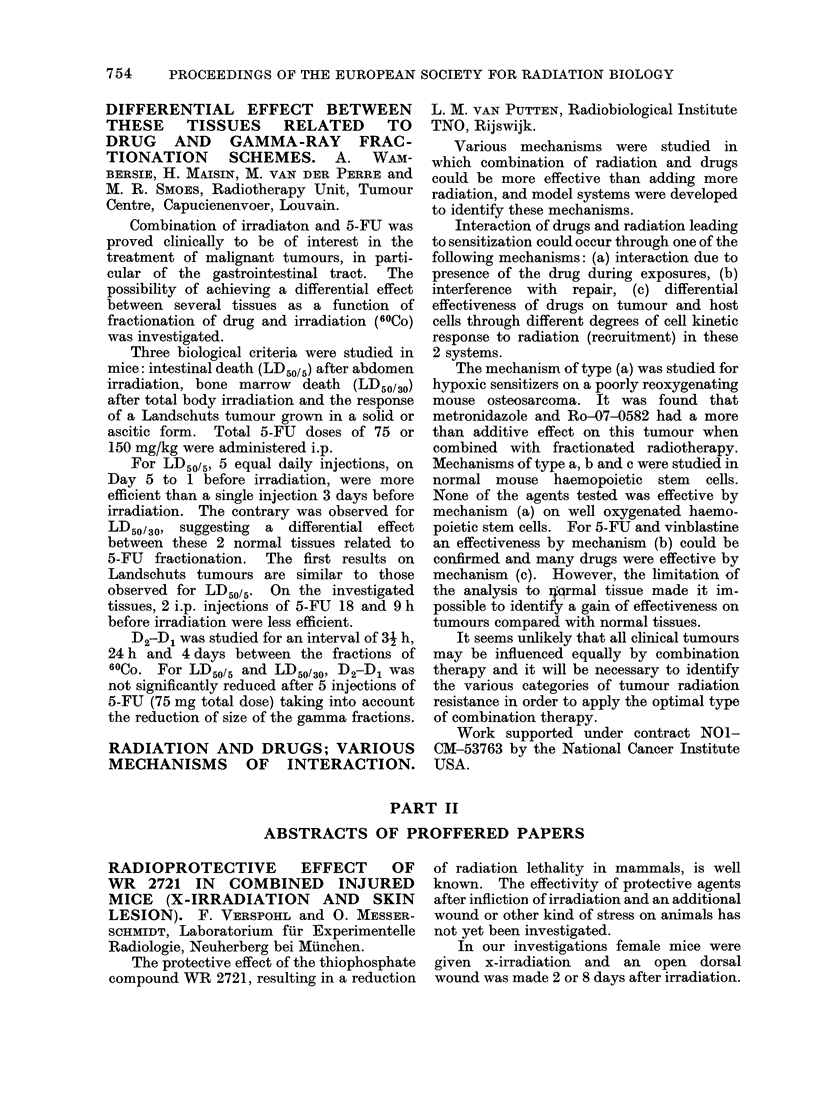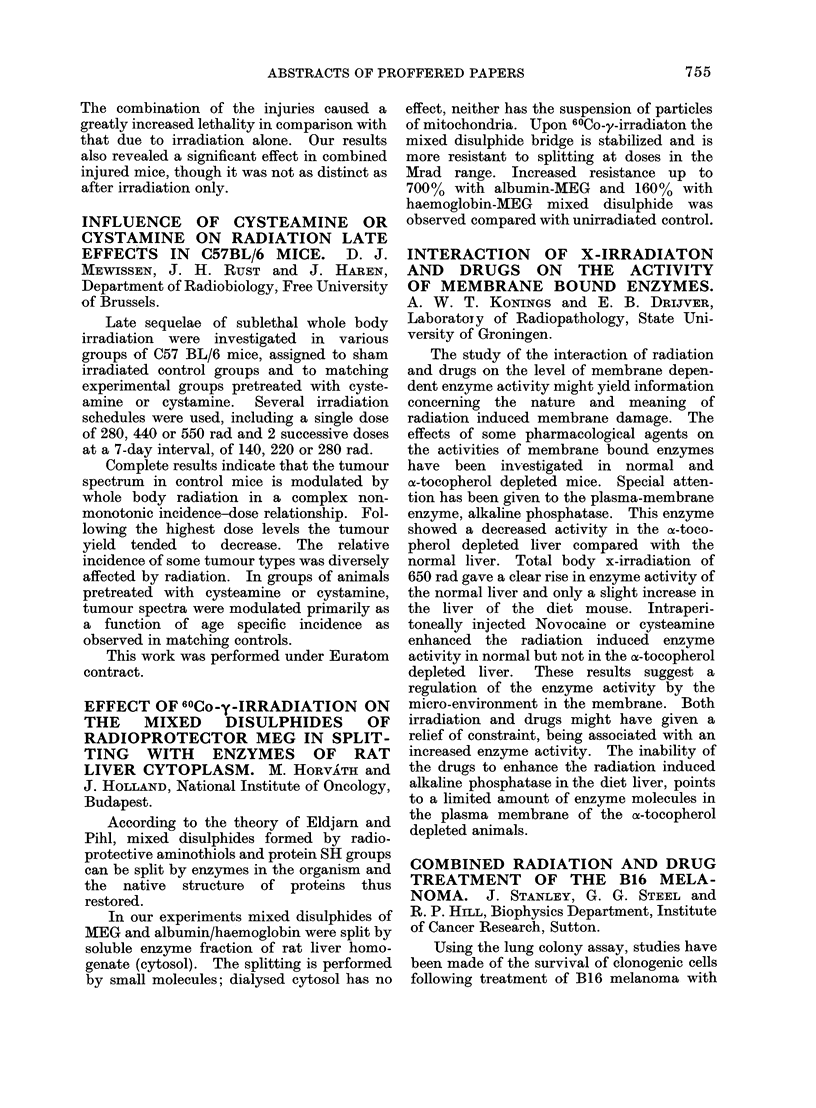# Proceedings: Radioprotective effect of WR 2721 in combined injured mice (x-irradiation and skin lesion).

**DOI:** 10.1038/bjc.1975.299

**Published:** 1975-12

**Authors:** F. Verspohl, O. Messerschmidt


### PART II

### ABSTRACTS OF PROFFERED PAPERS

**RADIOPROTECTIVE EFFECT OF WR 2721 IN COMBINED INJURED MICE (X-IRRADIATION AND SKIN LESION).** F. Verspohl and O. Messerschmidt, Laboratorium für Experimentelle Radiologie, Neuherberg bei München.

The protective effect of the thiophosphate compound WR 2721, resulting in a reduction of radiation lethality in mammals, is well known. The effectivity of protective agents after infliction of irradiation and an additional wound or other kind of stress on animals has not yet been investigated.

In our investigations female mice were given x-irradiation and an open dorsal wound was made 2 or 8 days after irradiation.

The combination of the injuries caused a greatly increased lethality in comparison with that due to irradiation alone. Our results also revealed a significant effect in combined injured mice, though it was not as distinct as after irradiation only.